# Ocular Accommodation, Intraocular Pressure, Development of Myopia and Glaucoma: Role of Ciliary Muscle, Choroid and Metabolism

**Published:** 2020-01-05

**Authors:** Karan R Gregg Aggarwala

**Affiliations:** Ocular Wellness and Nutrition Society, New York, United States

**Keywords:** Accommodation, Intraocular Pressure, Myopia, Glaucoma, Ciliary Muscle, Choroid

## Abstract

Ocular accommodation is not just a mechanism for altering curvature of the crystalline lens of the eye, it also enables aqueous humor outflow through the trabecular meshwork, influencing intraocular pressure (IOP). Long term stress on the ciliary muscle from sustained near focusing may initiate myopic eye growth in children and primary open angle glaucoma in presbyopic adults. Multi-factorial studies of ocular accommodation that include measures of IOP, ciliary muscle morphology, anterior chamber depth and assessment of nutritional intake and metabolic markers may elucidate etiology and novel strategies for management of both myopia and chronic glaucoma. Anatomy of the ciliary fibers from anterior insertion in the fluid drainage pathway to their posterior consanguinity with the vascular choroid, alters ocular parameters such as micro-fluctuations of accommodation and pulsatile ocular blood flow that are driven by cardiac contractions conveyed by carotid arteries. Stretching of the choroid has consequences for thinning of the peripheral retina, sclera and lamina cribrosa with potential to induce retinal tears and optic nerve cupping. Early metabolic interventions may lead to prevention or reduced severity of myopia and glaucoma. Finally, it might improve quality of life of patients and decrease disability from visual impairment and blindness.

## INTRODUCTION

Studies of the effect of short-term ocular accommodation on intraocular pressure (IOP) in the living human eye were published in the late 1950s and early 1960s at a time when continuous recording of IOP was regarded as a valuable tool for assessment of risk for glaucoma [1-3]. Etiology of normal tension glaucoma and primary open angle glaucoma is multifaceted and much uncertainty remains. Despite medical and surgical management and regular follow-up, about 14 to 16 percent of patients with chronic glaucoma become blind in one or both eyes within two decades of initial diagnosis [4]. 

In recent years, dynamic monitoring of changes in IOP in response to short-term accommodation has revealed a decline in baseline IOP (nearly 2 mm Hg) for progressing myopic as well as emmetropic young adults, for an accommodative demand of 3 diopters following 2 minutes of near fixation [5]. In the same study, in addition to a decline in baseline IOP, the ocular pulse amplitude (OPA) also declined in the both groups, and myopic subjects had a lower baseline OPA compared to emmetropic subjects, accompanied by a smaller decline in OPA with near focusing. The significance of these ocular pulse findings for aqueous humor drainage, myopic progression and the onset of glaucoma are now under debate. Prior studies on short-term near focusing have consistently reported a decline in IOP suggesting incremental aqueous outflow via the trabecular meshwork (TM). A recent study [6] using accommodative demand from zero up to 6 D has found transient elevation of IOP in progressing myopes (about 1 mm Hg). This was accompanied by decreased anterior chamber depth, narrowing of the angle between the peripheral iris and cornea, and a thickening of the natural crystalline lens of the eye, in both progressing myopes and emmetropes. Pioneering research published in 1970 showed [7] that the mean IOP of myopes was nearly 1 mm Hg higher than that of emmetropes, and this was statistically significant. About two decades later, a longitudinal study (of 2 year duration) authored by Jensen in 1992 demonstrated that the rate of myopia progression appeared to be slower in myopes with lower IOP values (in 49 children, aged 9 to 12 years) than those with higher IOP readings, suggesting a role for IOP in progression of myopia [8].

In studies of the anatomy of accommodation by Fisher [9], it was suggested that the mechanics of accommodation are reversible, with no evidence of hysteresis in the eyeball preparation following a cycle of stress and strain. Fisher further pointed out that after 30 years of age the force of the anterior ciliary muscle during accommodation steadily rises to a maximum and then may decrease to its juvenile value by about 60 years of age, perhaps consistent with changes in electrical impedance [10]. The crystalline lens accumulates fiber cells and grows throughout life, becoming increasingly more difficult to deform [11, 12]. Recent data on the age of onset of presbyopia in Germany suggest onset in the fifth decade of life [13], and an age of about 45 years was indicated from early work by Donders and Duane in European subjects [14]. Data from the Indian subcontinent suggest an average age of onset closer to 35 years [14], with impairment of accommodation among diabetics more than controls [15].

As reviewed by Nesterov (1986), in studies published between 1900 and 1943, fluid transport was demonstrated from the anterior chamber not only to the conventional trabecular outflow pathway, but to suprachoroidal spaces in the ciliary body, posterior sclera and choroid [16]. Such pathways for aqueous humor are now termed as the “unconventional” outflow pathway. Tracer studies conducted by Anders Bill contributed to such understanding and corneal, iris and retinal routes were identified [17].

Estimates of the relative contribution of the uveoscleral drainage pathway to total aqueous humor outflow, range from 25% to 60%, especially in eyes of younger humans. The fraction of uveoscleral aqueous humor drainage is reported to represent about 35% of the total in young adults, and far less in older adults [18]. The state of contraction of the ciliary muscle modulates uveoscleral outflow, i.e. contraction reduces outflow while relaxation increases it. Consequently, changes in ciliary tone modulate relative contributions of trabecular and uveoscleral outflow routes, with associated histological changes in the extracellular matrix [16-20]. 

Nerve supply to anterior ocular aqueous fluid drainage structures enables regulation of IOP by feedback mechanisms that are poorly understood [21]. Inflammation of the anterior segment reduces density of collagen in the extracellular matrix of the ciliary muscle [22], contributing to increased uveoscleral outflow. Topical pilocarpine decreases the size of spaces between the ciliary bundles [23], reducing uveoscleral outflow. However, the pressure lowering effect of this alkaloid is produced by forces transferred to the elastic trabecular network in the angle between the iris root and peripheral cornea, affecting the endothelium of Schlemm’s canal and juxta-canalicular tissue [24]. Increased ciliary body thickness with increasing axial myopia [25] suggests that natural near accommodation sustained daily for several hours and extended for months, may decrease unconventional outflow and clinical epidemiology data [26] are published. 

Current methods for measuring unconventional outflow are not entirely reliable [17], and research toward development of more accurate procedures may facilitate better management of glaucoma suspects, patients with normal tension glaucoma, open angle glaucoma, and possibly other types involving changes in the anterior segment of the eye. Glycosaminoglycans, fibronectin, laminin and collagen are considered relevant to trabecular outflow, as well as to deposition of fibrotic plaques in the TM of possible metabolic origin [27]. 

Reports reveal that the thickness of the ciliary body as it relates to refractive error in both children and adults is important for its possible explication of mechanisms that lead to myopia or elevation of IOP or both. In 53 children aged 8 to 15 years, thicker ciliary body measurements were associated with myopia and a longer axial length [28]. The relaxed ciliary muscle in older adults has an appearance similar to the accommodated ciliary muscle of a young individual [29], and the aged ciliary muscle may be less able to implement relaxation of accommodation [30], a sort of hysteresis, with unexplored consequences for aqueous drainage. The ciliary ring diameter and inner apex position are subject to change, mediated in part by tension in zonular fibers [31].

Thicker ciliary body morphology is associated with suppressed high-frequency micro-fluctuations of accommodation [32]. High frequency and low frequency fluctuations in accommodation are considered concomitant with pulsatile changes in IOP, although a visual information processing role including retinal control of eye focus is postulated. Decline in the amplitude of accommodative microfluctuations and pulsations of IOP with increased ciliary body thickness may be factors associated with ocular hypertension and stimulating myopic eye growth. A study of intraocular pulsations of IOP alongside laser interferometric measurements of cardiac synchronous fundus pulsations demonstrated a high association between fundus pulsation amplitude and tonometric pulse amplitude [33], suggesting a role for such pulsations in choroidal perfusion. The anatomical basis for such a connection lies in the posterior consanguinity (intimate connection) of the ciliary muscle with the choroid. 

Recent reports postulate that glaucoma, myopia and presbyopia are linked by anatomical adherence, elasticity, and force transfer properties of the choroid, and damage to the optic nerve may be caused in part by transient changes (spikes) in accommodation, IOP and choroidal tension [34, 35]. Posterior attachments of the ciliary muscle to the choroid pull the entire choroid and the adhering retina anteriorly by about 1 mm at the ora serrata during accommodation. Choroid biomechanics during accommodation have not been studied in living human eyes. How much tension is placed on the optic nerve head, particularly the lamina cribrosa during contraction of the posterior ciliary muscle in the living eye remains a matter of speculation. Physiological as well as glaucomatous cupping (excavation) of the optic nerve head may be in part due to choroidal biomechanics, which continue to pull on the posterior eyeball structures during accommodation, despite low to normal fluid eye pressure, and maybe after surgical removal of the crystalline lens and implant of a synthetic intra-ocular lens (IOL) to manage visual loss from cataract. Further, the role of such forces and retinal stretching in development of retinal tears, with possibility of progression to retinal detachment, particularly in axial myopia, has not been investigated in detail.

Survival of neurons, in general, depends on trophic factors released by target tissue innervated by those neurons during development [36]. The posterior uvea (choroid), contains smooth muscle, fibroblasts and endothelial cells, that provide molecular mechanisms for survival of ciliary ganglion neurons [37, 38]. Future studies of the mechanisms involved in such trophic activity, with their associated biochemical, hormonal and dietary precursors, are likely to aid development of neuro-protective strategies amenable to eye care practitioners, family physicians and public health professionals. 

The mechanisms controlling development and progression of myopia and chronic glaucoma appear to be related and there is substantial evidence for increased risk for primary open-angle glaucoma not just in moderate and high myopia, but also in low myopia [39]. Ergonomic factors, ambient light (spectrum and intensity) and physiological factors underlying eye growth for myopic refractive change [40], may potentially influence the onset of glaucoma [41], as well as stretching, atrophy and degeneration of the peripheral retina. A proposed difference between glaucoma and axial myopia could be the elastic properties of the sclera [42], with potential modifiable biochemical components [43].

Whereas several mechanisms have been proposed to relate myopia and glaucoma, well-substantiated evidence is lacking. Contraction of the longitudinal ciliary muscle and its effect on choroidal tension and stretching of the sclera suggest significance for the development of myopia [44]. In addition, biochemical and hormonal mechanisms that regulate growth factors in the choroid [45] and nutritional factors that influence IOP and ciliary muscle physiology [46, 47] warrant further investigation. Documentation of ciliary muscle backscatter and hysteresis [48] and near focusing induced reduction in depth of the anterior chamber following lens thickening and forward displacement [49], need to be correlated with nutritional, biochemical, metabolic, hormonal and environmental variables, as well as IOP and aqueous drainage. Such investigations require cooperation between professionals from diverse disciplines, and pose a challenge to the discrete, non-communicating domains (silos) of grant funding categories classified by agencies such as the Department of Health and Human Services and the National Institutes of Health.

## CONCLUSION

The onset and progression of myopia (near sightedness or defocused distance vision) in children and development of chronic open angle and normal tension glaucoma in middle aged adults have a common underlying feature as “sustained stress on the ocular accommodative mechanism.” Myopia and chronic glaucoma have high burdens for nations that are poorly equipped with trained personnel and economic resources, as well as for countries that have a higher gross domestic product. It is hoped that the present investigation stimulates additional researches supported by their host institutions. Early metabolic interventions may lead to prevention or reduced severity of myopia and glaucoma, as they are indicated for cataract [50]. Finally, such preventive strategies might improve quality of life and work productivity of patients and decrease disability from visual impairment and blindness, substantially reducing national expenditure on healthcare. Medical ophthalmologists, clinical optometrists, pediatricians, family physicians, naturopathic doctors, functional and environmental medicine doctors, endocrinologists, nurses, epidemiology researchers, national agencies that impact science, food supply and public health (e.g. the DHHS, NIH, FDA, CDC, USDA, and ARS) and those associated to the United Nation (e.g. WHO, FAO, UNESCO, UNICEF), can all potentially cooperate in this endeavor. 
